# Resource availability and parasitism intensity influence the response of soybean to the parasitic plant *Cuscuta australis*

**DOI:** 10.3389/fpls.2023.1177154

**Published:** 2023-05-09

**Authors:** Yong-Ge Yuan, Fang-Lei Gao, Fei-Hai Yu, Jun-Min Li, Mai-He Li

**Affiliations:** ^1^ School of Advanced Study, Taizhou University, Taizhou, China; ^2^ Zhejiang Provincial Key Laboratory of Plant Evolutionary Ecology and Conservation, School of Life Sciences, Taizhou University, Taizhou, China; ^3^ Shandong Key Laboratory of Eco-Environmental Science for the Yellow River Delta, Binzhou University, Binzhou, China; ^4^ Swiss Federal Institute for Forest, Snow and Landscape Research WSL, Birmensdorf, Switzerland; ^5^ Key Laboratory of Geographical Processes and Ecological Security in Changbai Mountains, Ministry of Education, School of Geographical Sciences, Northeast Normal University, Changchun, Jilin, China; ^6^ College of Life Science, Hebei University, Baoding, Hebei, China

**Keywords:** parasite tolerance, phosphorus availability, water availability, growth, resource allocation

## Abstract

**Introduction:**

Parasitic plants can damage crop plants and consequently cause yield losses and thus threaten food security. Resource availability (e.g., phosphorus, water) has an important role in the response of crop plants to biotic attacks. However, how the growth of crop plants under parasitism are affected by environmental resource fluctuation is poorly understood.

**Methods:**

We conducted a pot experiment to test the effects of the intensity of *Cuscuta australis* parasitism and the availability of water and phosphorus (P) on soybean shoot and root biomass.

**Results and discussion:**

We found that low-intensity parasitism caused ~6% biomass reduction, while high-intensity parasitism caused ~26% biomass reduction in soybean. Under 5–15% water holding capacity (WHC), the deleterious effect of parasitism on soybean hosts was ~60% and ~115% higher than that under 45–55% WHC and 85–95% WHC, respectively. When the P supply was 0 μM, the deleterious effect of parasitism on soybean was 67% lower than that when the P supply was 20 μM. Besides, the biomass of *C. australis* was highest when both the water and the P availability were lowest. *Cuscuta australis* caused the highest damage to soybean hosts under 5 μM P supply, 5–15% WHC, and high-intensity parasitism. Additionally, *C. australis* biomass was significantly and negatively related to the deleterious effect of parasitism on soybean hosts and to the total biomass of soybean hosts under high-intensity parasitism, but not under low-intensity parasitism. Although high resource availability can promote soybean growth, the two resources have different impacts on the response of hosts to parasitism. Higher P availability decreased host tolerance to parasites, while higher water availability increased host tolerance. These results indicate that crop management, specifically water and phosphorus supply, can efficiently control *C. australis* in soybean. To our best knowledge, this appears to be the first study to test the interactive effect of different resources on the growth and response of host plants under parasitism.

## 1 Introduction

As a unique group of angiosperms, parasitic plants have evolved the strategy of directly absorbing nutrients and water from their host plants through haustoria (Press and Phoenix, 2005). Parasitic plants can inhibit the growth of host plants and consequently impact community structure and even the functioning of ecosystems, especially agroecosystems (Press and Phoenix, 2005; Bardgett et al., 2006; Pointurier et al., 2021). It has been reported that parasitic plants are one of the major threats to the cultivation of crops, as they can pose a deleterious impact on crop production (Tarr, 1957; Olowe et al., 2023). For example, the parasite *Cuscuta* can cause 30-50% soybean (*Glycine max* (L.) Merr.) production loss in Henan province, China (Zhao, 2010). After parasitizing host plants, parasitic plants can induce a trade-off between growth and defense (Runyon et al., 2008). Therefore, exploring the factors affecting the performance of crop host plants to parasitic plants is of great importance in finding effective ways to control parasites.

Resources, such as water, nutrients, and light, are important for the growth and development of plants (Kozlowski and Pallardy, 1997; Kozlowski and Pallardy, 2002; Salazar-Mendoza et al., 2023). With changes in resources availability, plants exhibit morphological, physiological, and biochemical changes, consequent changes in metabolites, enzymes, and behaviors, and ultimately adaptive changes in phenotype and genotype (Vierling and Kimpel, 1992; Salazar-Mendoza et al., 2023). The physiological and biochemical changes in host plants can induce ecologically important responses of parasitic plants (Evans and Borowicz, 2015; Zhang et al., 2023) and result in indirect effects of host plants on the growth of parasitic plants (Zagorchev et al., 2021). For example, fertilization can boost the growth of parasitic plants and thus enhance the deleterious effects of parasitic plants on host plants (Yang et al., 2015). In contrast, Miller et al. (2003) found that parasitic *Amyema miquelii* infection increased as the water stress of host *Eucalyptus largiflorens* decreased, while Evans and Borowicz (2013) found that drought stress enhanced damage from parasitic *Cuscuta gronovii* to host *Verbesina alternifolia*. However, the effect of resource availability on the tolerance of host plants to parasitism remains unclear.

Plants always confront multiple limiting environmental resources (Ho et al., 2005; Lankford et al., 2023). Water and phosphorus (P) are essential to plants (He et al., 2017), and they are notable for representing extremes of contrasting resource availability, i.e., water is ephemeral and mobile, whereas P is stable and immobile (Ho et al., 2005). P is a scarce and non-renewable resource (Gilbert, 2009; de Goes et al., 2023), and drought is expected to increase (both in frequency and severity) in the future, as a consequence of decreased regional precipitation or increased evaporation driven by global warming (Sheffield et al., 2012). Drought restricts soil P diffusion and P uptake in plants (Suriyagoda et al., 2014), while P fertilization increases water use efficiency (Payne et al., 1992) and drought tolerance (Jones et al., 2005) and alleviates the impact of drought on plant yield (Jones et al., 2003). Drought and low P availability are two important factors that limit the yield of plants (Manavalan et al., 2009; de Goes et al., 2023), and the interactive effect of water and P acquisition on plant growth has been previously described (Ho et al., 2005; He et al., 2017). Until now, little has been known about the effects of water and P availabilities on the response of host plants to parasitic plants.

There are two hypotheses regarding how tolerance of hosts to herbivory is affected by resource conditions, i.e., the growth rate model (GRM) (Hilbert et al., 1981; Wise and Abrahamson, 2007) and the compensatory continuum hypothesis (Maschinski and Whitham, 1989; Long and Porturas, 2014; Venter et al., 2021). GRM predicts lower tolerance of herbivore damage in higher-resource environments, while the compensatory continuum hypothesis predicts greater tolerance of herbivory damage in higher-resource environments. Although herbivores and parasitic plants possess similar feeding preferences (Marquardt and Pennings, 2010; Gao et al., 2019). However, so far, no studies have tested these two hypotheses on parasitic plants.

The intensity of parasitic plant parasitism on host plants has been confirmed to quantitatively affect the growth of host plants and induce host plants to utilize compensatory growth strategies (Zhang et al., 2012). Such different levels of stress from parasitism, herbivory, or grazing may result in different effects of resource availability on the growth or defense of host plants. For example, Gao et al. (2008) showed significant interactions between water availability and simulated grazing gradient on the relative high growth rate and bud number in the wild rye, *Leymus chinensis*. However, no interactive effects of parasitism intensity with water and P availability have been examined from the perspective of the growth of host plants and their tolerance to parasitic plants.

In this study, we conducted a common pot experiment to test the effect of water and P availability together with parasitism intensities on the growth of holoparasitic *Cuscuta australis* and host soybean. *C. australis* is a holoparasitic plant that feeds on the stems of plants and is known to acquire water, carbon, and nutrients from its host *via* haustoria, and heavily suppresses the growth of its host plant in the field (Zhang et al., 2012; Li et al., 2015). The life cycle of *C. australis* includes: (1) the seed germination; (2) the early development of the seedling; (3) the search for a host plant; (4) and the interaction with the host plant (Yoder, 1999; Furuhashi et al., 2011). We hypothesized that (1) both water availability and P availability would increase soybean host biomass, *C. australis* biomass, and the deleterious effect of *C. australis* on soybean hosts; (2) heavy parasitism would decrease soybean host biomass, but increase both *C. australis* biomass and the deleterious effect of *C. australis* on soybean hosts; and (3) there are synergetic effects between water availability and P availability that are associated with parasitism intensity. These results would provide informed scientific bases for the control of parasitic weeds in agroecosystems.

## 2 Materials and methods

### 2.1 Plant materials

Soybean seeds (*Glycine max* (Linn.) Merr.) of Zhonghuang 37 were used as host plants in this study. They were cultivated by the Chinese Academy of Agricultural Sciences, Beijing, China. Soybean plants are common hosts for *Cuscuta* parasitic species. In agricultural systems, the growth and development of soybeans are significantly suppressed by *Cuscuta* parasitism (Zhao, 2010). Three soybean seeds were sowed (3 cm deep) in each of 315 pots (18 cm in height, 10 cm in diameter) filled with 1.5 kg of quartz sand each. Quartz sand was used as the substrate because it contained minor amounts of P. As such, we could easily control the P concentration, as suggested by Li et al. (2019). After germination, each pot was thinned to one healthy seedling for the experiment.

### 2.2 Experiment design

The pot experiment with soybean as the target plant had a 3 × 5 × 3 factorial design with three levels of parasitism (no parasitism and low- and high-intensity parasitism), five levels of P availability, and three levels of water availability. Each treatment combination had seven replicates, for a total of 315 pots.

When the soybean seedlings were approximately 15 cm tall, different combinations of parasitism, P, and water availability treatments were applied. First, one-third out of the 315 pots (i.e., 105 soybean plants) were randomly selected and treated with one of the three levels of parasitism treatments by winding zero, one, or three 15-cm-long precultured *C. australis* segments around soybean stems to represent no parasitism and low- and high-intensity parasitism (C0, C1, C2), respectively. At the same time, one-fifth of the pots (i.e., 21 soybean plants) within each parasitism level were randomly assigned to be treated with one of the five P levels, i.e., 0 (P0), 5 (P5), 10 (P10), 15 (P15), and 20 (P20) μM per pot. P was supplied as KH_2_PO_4_ solution based on Hoagland’s formula (Hoagland and Arnon, 1950). Macro-elements (nitrogen, potassium, magnesium, calcium) were also included in the Hoagland solution. All pots were supplied with the same amounts of macro-elements, except for P. Meanwhile, one-third of those 21 pots (i.e., 7 soybean plants) were randomly selected for each of three water treatment levels according to water holding capacity (WHC), i.e., 5–15% (W1), 45–55% (W2), and 85–95% (W3) WHC. The soil water content was monitored using Soil Survey Instrument 4 in 1 Ituin (PT Biruni Geo Pratama, Banten, Indonesia).

### 2.3 Measurements and analysis

Eight weeks after the treatments began, target plants with five to seven replicates (*n* = 5 to 7) for each treatment combination were harvested, as replicate pots in which plants died by the harvest date were excluded from measurement and analysis. The *C. australis* plants were also harvested by detaching them from soybean hosts. Next, the soybean plants were separated into shoots and roots by cutting the shoot at a 2-cm stem height above the soil surface. Then, the shoot and root biomass was oven-dried at 65°C to a constant weight.

The root/shoot ratio (R/S ratio) was calculated as the ratio of root biomass to shoot biomass. The deleterious effect (DE) was calculated as the difference in total biomass between parasitized plants and the mean total biomass of the unparasitized plants, standardized by the mean total biomass of the unparasitized plants within each treatment (Barton, 2008; Li et al., 2012). The lower the DE value, the stronger the negative effect of *C. australis* on the host soybean and the lower the tolerance of soybean to parasitism (Barton, 2008; Li et al., 2012).

### 2.4 Statistical analysis

We used three-way ANOVAs to examine the effects of parasitism (C0, C1, C2), P availability (P0, P5, P10, P15, P20), and water availability (W1, W2, W3) on host soybean biomass (total biomass and R/S ratio). We also used three-way ANOVAs to examine the effects of parasitism level (C1, C2), P supply (P0, P5, P10, P15, P20), and water supply (W1, W2, W3) on *C. australis* biomass and on the deleterious effect of *C. australis* on soybean. Before the statistical analysis, assumptions of normality and homogeneity of variance were assessed based on standardized residual analysis. Data were transformed, if necessary, using the appropriate transformations, specifically natural logarithm (ln) transformation for the R/S ratio and for biomass of *C. australis*. Moreover, a simple linear regression model (*y* = *a* + *bx*) was used to fit a straight line to the relationships between the biomass of the parasitic plant *C. australis* and the deleterious effect of *C. australis* on soybean, and between the biomass of *C. australis* and total biomass of soybean under low- or high-intensity parasitism. Additionally, a simple linear regression model (*y* = *a* + *bx*) was also used to fit a straight line to the relationship between water availability and the deleterious effect of *C. australis* on soybean when the P supply was 0, 5, or 10 μM. To determine whether the slopes of the regression lines were significantly different between different P availabilities, the homogeneity of the slopes (i.e., parallelism) was tested *via* one-way ANCOVA (Du et al., 2016). Parallel regression lines indicated that the deleterious effect in different P availabilities in response to water availabilities was identical, while the non-parallel regression lines indicated that the deleterious effect in different P availabilities in response to water availabilities differed. We used structural equation modelling (SEM) to analyze hypothetical pathways that may explain how changes in P availability, water availability, and parasitism directly or indirectly affect the total biomass of soybean and the biomass of *C. australis*. The SEMs were implemented using the LAVAAN package. The data were analyzed in R v.3.5.0 through the RStudio platform (R Core Team, 2018).

## 3 Results

### 3.1 Growth of host plant soybean

Parasitism by *C. australis*, P availability, and water availability significantly affected the total biomass of soybean plants (**Table 1**). Specifically, parasitism by *C. australis* significantly decreased the total biomass of soybean plants, and high-intensity parasitism (C2) significantly decreased the total biomass of soybean (~26%) relative to low-intensity parasitism (C1) (~6%) (**Figure S1A**). The total biomass of soybean plants under 5–15% WHC (W1) was significantly lower than that under both 45–55% WHC (W2) and 85–95% WHC (W3) (14% and 12%, respectively) (**Figure S1B**). The total biomass of soybean plants reached its maximum when P availability was 15 μM (P15). There was no significant difference in the total biomass of soybean when the P supply was 10, 15, and 20 μM. When the P supply was 20 μM, the total biomass of soybean was 32% higher than that when the P supply was 0 μM (**Figure S1C**).

**Table 1:** Results of three-way ANOVAs testing the main effects and interactions of *Cuscuta australis* parasitism (C), P availability (P), and water availability (W) on growth of soybean hosts.

	variables	*d.f.*	*F* value	*p* value
Total biomass	C	2	56.668	**<0.001**
	P	4	25.310	**<0.001**
	W	2	15.575	**<0.001**
	C*P	8	1.541	0.143
	C*W	4	1.655	0.161
	P*W	8	2.272	**0.023**
	C*P*W	16	2.406	**0.002**
Shoot biomass	C	2	68.464	**<0.001**
	P	4	29.005	**<0.001**
	W	2	7.557	**<0.001**
	C*P	8	1.524	0.149
	C*W	4	2.118	0.079
	P*W	8	1.797	0.078
	C*P*W	16	2.251	**0.004**
Root biomass	C	2	8.093	**<0.001**
	P	4	5.396	**<0.001**
	W	2	35.492	**<0.001**
	C*P	8	1.463	0.171
	C*W	4	0.927	0.449
	P*W	8	2.578	**0.010**
	C*P*W	16	2.036	**0.012**
R/S ratio	C	2	51.0823	**<0.001**
	P	4	13.4806	**<0.001**
	W	2	5.8553	**0.003**
	C*P	8	1.0966	0.366
	C*W	4	3.7333	**0.006**
	P*W	8	0.9458	0.479
	C*P*W	16	1.5739	0.076

Values are in bold when *p* < 0.05.

**Figure 1:**
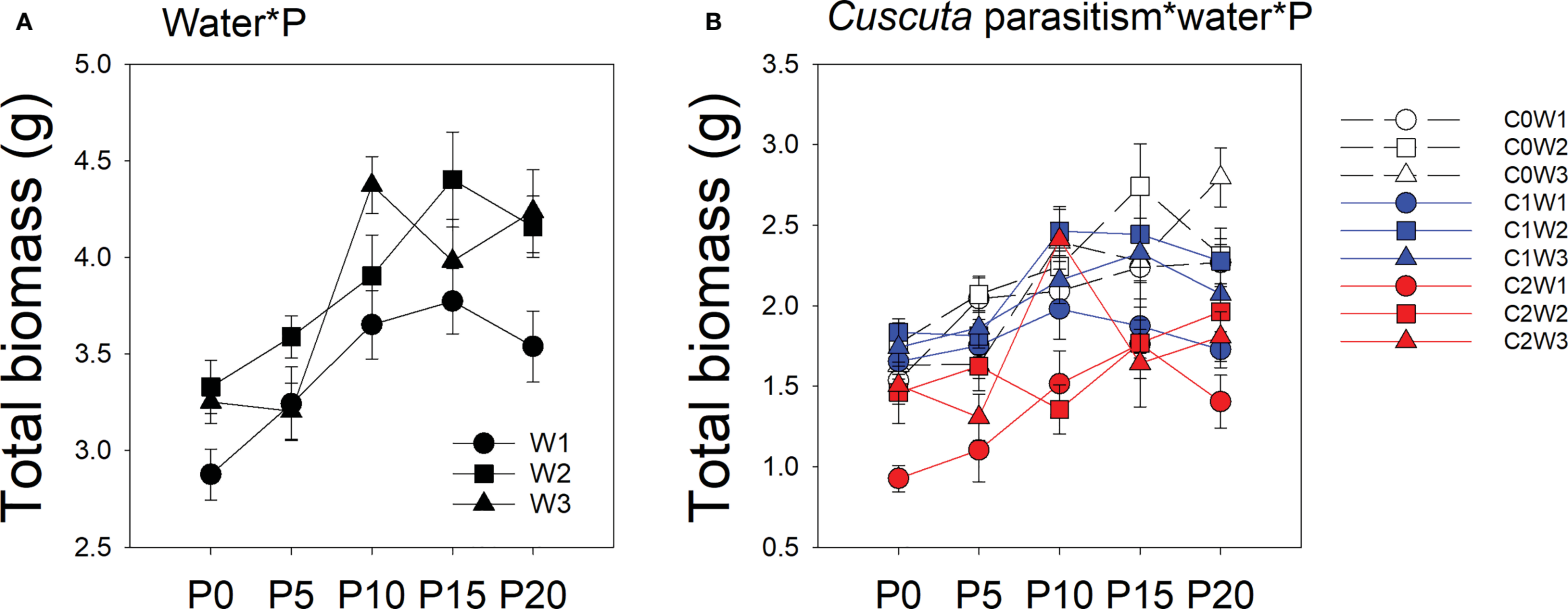
Mean ( ± SE) total biomass of soybean host plants. The panels show significant effects of **(A)** the interaction between water availability and P availability; **(B)** the interaction between parasitism level, water availability, and P availability. C0, C1, and C2 indicate no parasitism and low- and high-intensity parasitism by *C. australis*, respectively. W1, W2, and W3 indicate that soil water content was 5–15%, 45–55%, and 85–95% water holding capacity, respectively. P0, P5, P10, P15, and P20 indicate P availability levels of 0, 5, 10, 15, and 20 μM, respectively.

There was a significant interaction between water availability, P availability, and parasitism intensity (**Table 1**; **Figure 1**). Specifically, under low-intensity parasitism, when water availability was 5–15% WHC (W1), the total biomass of soybean was less changed with P availability, while when water availability was 85–95% WHC (W3), the total biomass of soybean showed an increasing trend with P availability. Under high-intensity parasitism and 5–15% WHC (W1), the total biomass of soybean first increased as P supply increased and then slightly decreased, though not significantly (**Figure 1**). However, under high-intensity parasitism and 85–95% WHC (W3), there was a fluctuation in total biomass of soybean when the P supply was 5–15 μM (P5–P15) (**Figure 1**).

**Figure 2:**
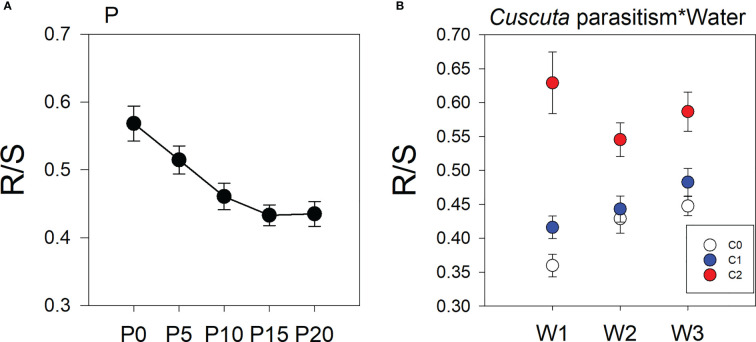
Mean ( ± SE) the root/shoot (R/S) ratio of soybean host plants. The panels show significant effects of **(A)** the main effect of P availability across five levels; **(B)** the interaction between parasitism and water availability. C0, C1, and C2 indicate no parasitism and low- and high-intensity parasitism by *C. australis*, respectively. W1, W2, and W3 indicate that soil water content was 5–15%, 45–55%, and 85–95% water holding capacity, respectively. P0, P5, P10, P15, and P20 indicate P availability levels of 0, 5, 10, 15, and 20 μM, respectively.

Treatments of parasitism, P availability, and water availability had a significant influence on the R/S ratio of soybean plants (**Table 1**). Specifically, parasitism by *C. australis* significantly increased the R/S ratio of soybean plants. Under high-intensity parasitism (C2), the R/S ratio of soybean plants was 43% higher than that under no parasitism, and 31% higher than that under low-intensity parasitism(**Figure S2A**). As water availability increased, the R/S ratio of soybean plants increased (**Figure S2B**), while the R/S ratio of soybean plants decreased with the increasing P availability (**Figure 2A**). There was a significant interaction between parasitism and water availability in the R/S ratio of soybean plants (**Table 1**). Under no parasitism or low-level parasitism, the R/S ratio of soybean plants increased with water availability, while under heavy-level parasitism, the R/S ratio of soybean plants was lowest under 45–55% WHC (W2) (**Figure 2B**).

### 3.2 Growth of the parasitic plant *Cuscuta australis* and its deleterious effect on soybean hosts

**Figure 3:**
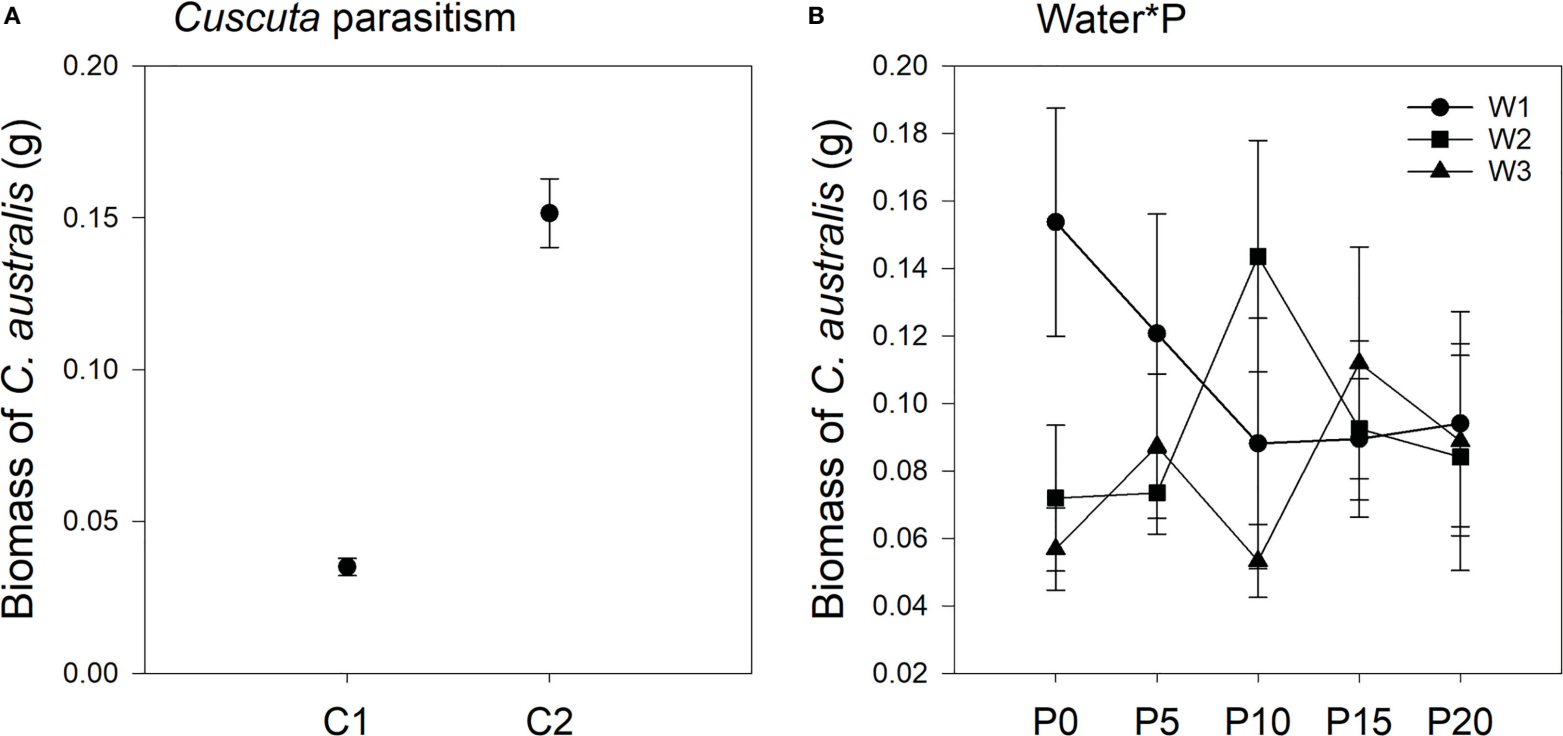
Mean ( ± SE) biomass of the parasitic plant *Cuscuta australis*. The panels show significant effects of **(A)** the main effect of parasitism by *Cuscuta australis*; **(B)** the interaction between water availability and P availability. C1 and C2 indicate low- and high-intensity parasitism by *C. australis*, respectively. W1, W2, and W3 indicate that soil water content was 5–15%, 45–55%, and 85–95% water holding capacity, respectively. P0, P5, P10, P15, and P20 indicate P availability levels of 0, 5, 10, 15, and 20 μM, respectively.

Parasitism level significantly affected the biomass of *C. australis* (**Table 2**; **Figure 3**). Under high-intensity parasitism (C2), the biomass of *C. australis* was 333% higher than that under low-intensity parasitism (C1) (**Figure 3A**). There was a significant interaction between P availability and water availability on the biomass of *C. australis* (**Table 2**; **Figure 3B**). Under 5–15% WHC (W1), the biomass of *C. australis* was first decreased and then unchanged as P availability increased. Under 45–55% WHC (W2), the biomass of *C. australis* was highest when P availability was 10 μM (P10). Under 85–95% WHC (W3), the biomass of *C. australis* did not significantly change as P availability increased (**Figure 3B**).

**Table 2:** Results of three-way ANOVAs testing the main effects and interactions of *Cuscuta australis* parasitism intensity (CL), P availability (P), and water availability (W) on *C. australis* biomass and on the deleterious effect of *C. australis* parasitism to soybean hosts.

	variables	*d.f.*	*F* value	*p* value
Biomass of *C. australis*	CL	1	198.220	**<0.001**
	P	4	0.633	0.640
	W	2	1.816	0.166
	CL*P	4	0.500	0.736
	CL*W	2	0.053	0.949
	P*W	8	2.813	**0.006**
	CL*P*W	8	1.024	0.419
Deleterious effect	CL	1	63.297	**<0.001**
	P	4	4.314	**0.002**
	W	2	7.542	**0.001**
	CL*P	4	1.083	0.367
	CL*W	2	1.227	0.296
	P*W	8	2.802	**0.006**
	CL*P*W	8	3.463	**0.001**

Values are in bold when p < 0.05.

**Figure 4:**
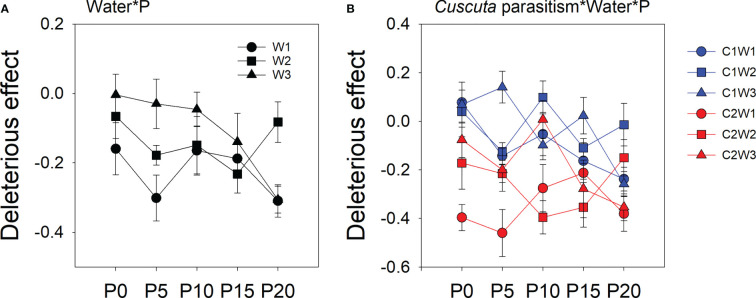
Mean ( ± SE) deleterious effect of *Cuscuta australis* on soybean host plants. The panels show significant effects of **(A)** the interaction between water availability and P availability; **(B)** the interaction between parasitism, water availability, and P availability. C1 and C2 indicate low- and high-intensity parasitism by *C. australis*, respectively. W1, W2, and W3 indicate that soil water content was 5–15%, 45–55%, and 85–95% water holding capacity, respectively. P0, P5, P10, P15, and P20 indicate P availability levels of 0, 5, 10, 15, and 20 μM, respectively.

The deleterious effect of *C. australis* on soybean plants was significantly affected by parasitism level, P availability, and water availability (**Table 2**). Specifically, under high-intensity parasitism (C2), the deleterious effect on the growth of soybean plants was 425% higher than that under low-intensity parasitism (C1) (**Figure S3A**). As water availability increased, the deleterious effect of *C. australis* on soybean plants decreased (**Figure S3B**), while as P availability increased, the deleterious effect of *C. australis* on soybean plants increased (**Figure S3B**). There was a significant interaction between water availability, P availability, and parasitism intensity in the deleterious effect of *C. australis* on soybean plants (**Table 2**). Specifically, under low-intensity parasitism, when water availability was 5–15% WHC (W1) and 85–95% WHC (W3), the deleterious effect of parasitism on soybean showed an overall increasing trend with P availability. Under high-intensity parasitism, when water availability was 5–15% WHC (W1), the deleterious effect of *C. australis* on soybean plants first decreased and then increased with P availability. When water availability was 45–55% WHC (W2), the deleterious effect of *C. australis* on soybean plants first increased and then decreased with P availability. When water availability was 85–95% WHC (W3), the deleterious effect of *C. australis* on soybean plants decreased slightly at the beginning and then increased with P availability (**Figure 4**). *C. australis* caused the highest deleterious effect to soybean hosts under 5 μM P supply, 5–15% WHC, and high-intensity parasitism.

### 3.3 Correlation between parasitic damage to soybean plants and *Cuscuta australis* biomass

**Figure 5:**
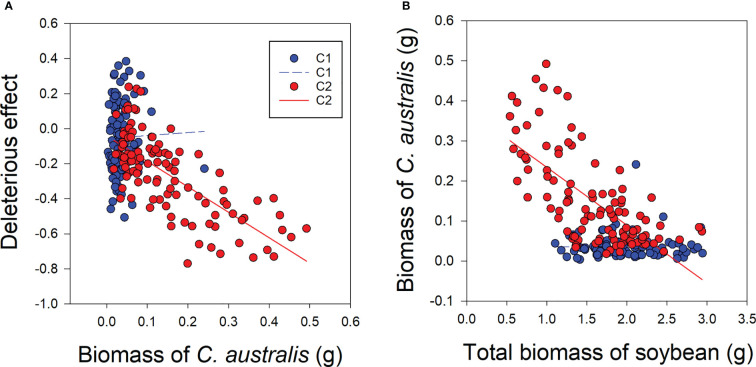
Correlations between biomass of the parasitic plant *Cuscuta australis* and the deleterious effect of *C*. *australis* on soybean host plants **(A)**, and between *C*. *australis* biomass and total biomass of soybean **(B)** under low- or high-intensity parasitism. C1 and C2 indicate low- and high-intensity parasitism by *C. australis*, respectively. The dashed line indicates a non-significant correlation, while the solid lines indicate significant correlations between *C*. *australis* biomass and the deleterious effect.

Under low-level parasitism (C1), there was no significant relationship between *C. australis* biomass and the deleterious effect of *C. australis* on soybean plants or between *C. australis* biomass and total soybean plant biomass (**Figures 5A, B**). However, under high-intensity parasitism (C2), *C. australis* biomass was significantly and negatively correlated with the deleterious effect of *C. australis* on soybean plants and also with the total soybean plant biomass (**Figures 5A, B**).

### 3.4 Correlation between water availability and the deleterious effect on soybean

**Figure 6:**
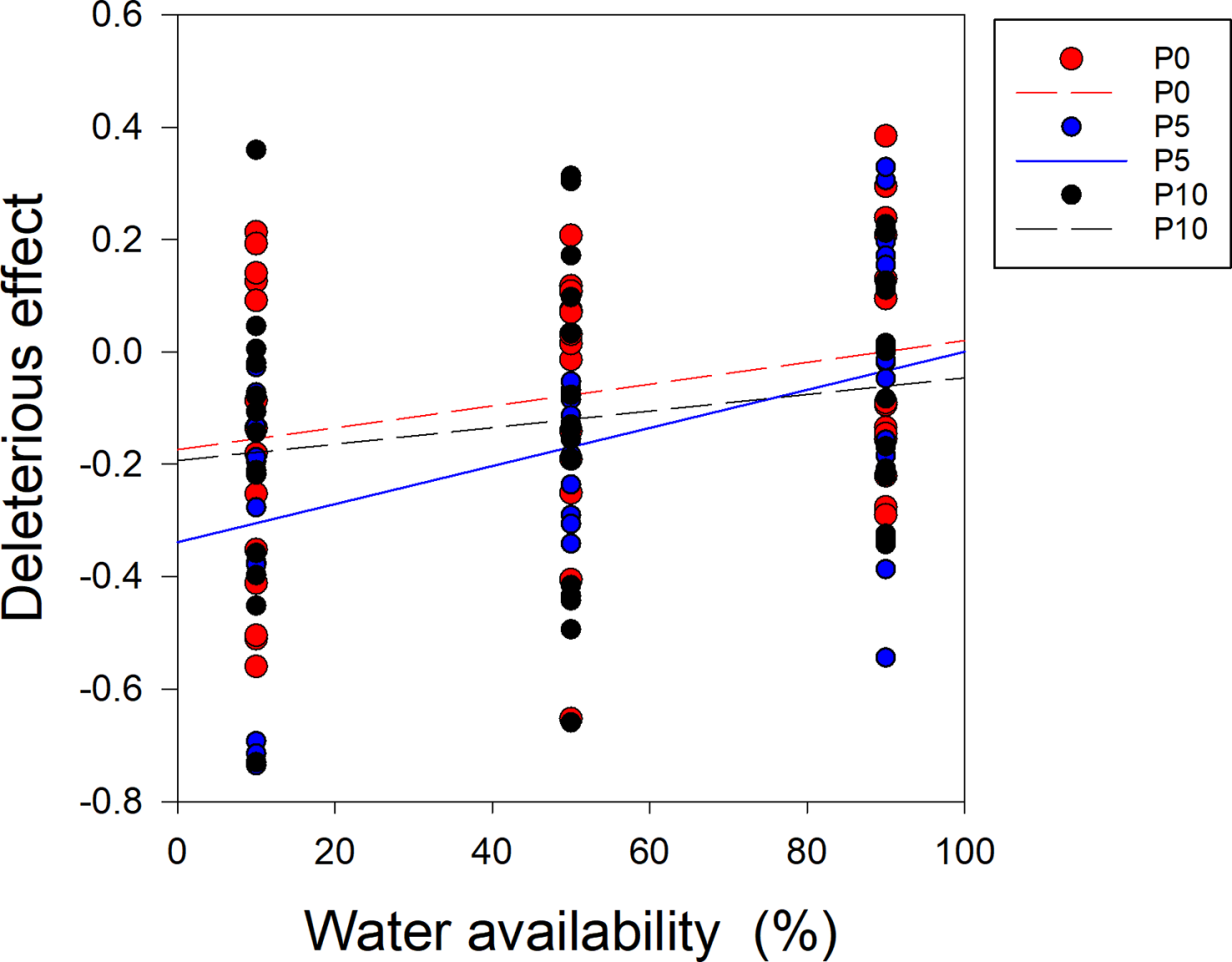
Correlations between the deleterious effect of *Cuscuta australis* on soybean host plants and water availability. The dashed line indicates a non-significant correlation, while the solid lines indicate significant correlations between water availability and the deleterious effect. P0, P5, and P10 indicate P availability levels of 0, 5, and 10 μM, respectively.

When the P supply was 0 and 10 μM (P0, P10), there was no significant relationship between water availability and the deleterious effect on soybean (**Figure 6**). However, when the P supply was 5 μM (P5), there was a significantly positive correlation between water availability and the deleterious effect on soybean (**Figure 6**). The slopes of the regression lines for water availability and the deleterious effect on soybean significantly differed between P5 and P10 (**Figure 6**).

### 3.5 Pathways determining soybean biomass and *Cuscuta australis* biomass

**Figure 7:**
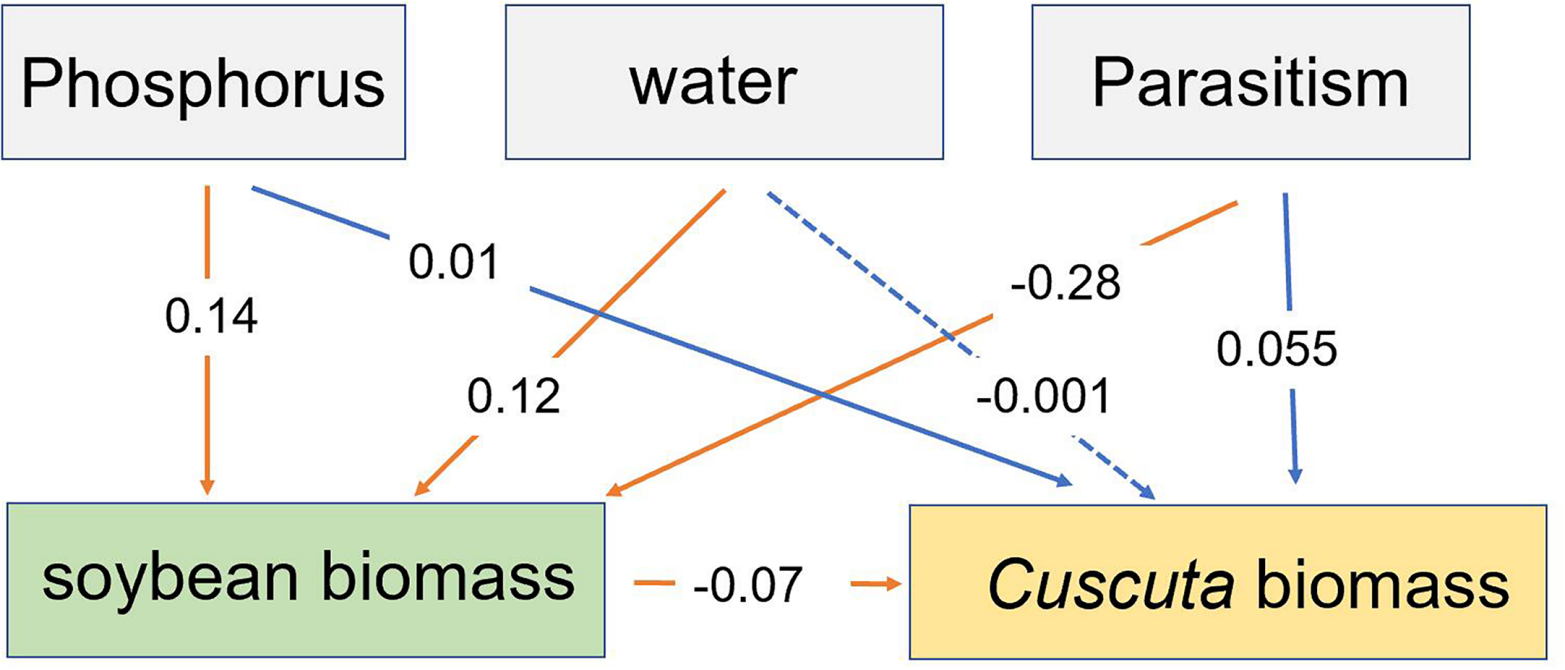
The structural equation model showing how the effect of P availability, water availability, and parasitism affected the total biomass of soybean and biomass of *Cuscuta australis*. Solid lines represent significant paths (*p* < 0.05), while the dotted line represents a non-significant path. Red lines represent direct paths, while blue lines represent indirect paths. Numbers near the lines show standardized regression weights.

Our SEM analysis showed that P availability and water availability directly increased soybean biomass, while parasitism directly decreased soybean biomass. The altered soybean biomass, in turn, decreased *C. australis* biomass (**Figure 7**). Additionally, P availability indirectly increased *C. australis* biomass, while water availability had a non-significant indirect effect on *C. australis* biomass (**Figure 7**).

## 4 Discussion

### 4.1 Effect of water availability

Several studies have shown that drought stress can inhibit the growth of host plants and consequently weaken the growth of parasitic plants (Miller et al., 2003; Evans and Borowicz, 2015). Evans and Borowicz (2013) also tested the impact of drought on the tolerance of *Verbesina alternifolia* to *C. gronovii* and found that drought stress enhanced the damage to host plants caused by parasitic plants. In our study, soybean plants under higher water availability grew more rapidly (**Figures S1B**, **7**), supporting less (under low P availability) or no change in (under high P availability) parasitic *C. australis* biomass (**Figure 3B**) as well as less damage to host plants caused by parasitic plants (**Figure S3B**). This can be explained by the compensatory continuum hypothesis (Maschinski and Whitham, 1989; Long and Porturas, 2014; Venter et al., 2021) and the plant stress hypothesis (White, 1969; Pardo and Pulido, 2017).

The compensatory continuum hypothesis predicts greater tolerance of herbivory damage in higher-resource environments (Maschinski and Whitham, 1989; Long and Porturas, 2014; Venter et al., 2021), while the plant stress hypothesis assumes that stress increases the availability of nutrients or decreases secondary metabolites, resulting in the increased impact of herbivores on plant fitness (White, 1969; Pardo and Pulido, 2017). Although these hypotheses refer to herbivory, we speculated that these hypotheses that explain plant response to herbivory may provide insight into the response of host plants to parasitism, as herbivores and parasitic plants possess similar feeding preferences (Marquardt and Pennings, 2010; Gao et al., 2019) and the interactions between parasitic plants and their hosts resemble herbivore–host interactions (Pennings and Callaway, 2002; Vaello et al., 2018). In our study, *C. australis* biomass was highest when both water and P resources availability were lowest (**Figure 3B**), and the deleterious effect on soybean was also highest when water resource ability was lowest. These results indicated that lower resource availability reduced the tolerance of soybean plants to parasitism, consistent with the plant stress hypothesis. Additionally, although the deleterious effect of *C. australis* to soybean plants was mediated by P availability, the deleterious effect was lowest when water availability was highest (under 85–95% WHC) (**Figure 4A**), consistent with the compensatory continuum hypothesis. Similar results were revealed by Lin et al. (2021) in their findings on the change in tolerance to herbivory of tomato (*Solanum lycopersicum*) under different levels of water availability.

The mechanism of how water availability affects the growth of host plants and parasitic plants remains unknown. Gassmann (2004) found that a higher R/S ratio might explain the higher tolerance of soybean plants under higher water availability by increasing the ability of roots to obtain resources. Increased R/S ratios can not only increase a plant’s stored carbon reserves and reallocation of carbon to above-ground biomass following damage (Xu et al., 2015), but also elevate the level of compensatory photosynthesis by increasing the supply of water and nitrogen to leaf tissue (McNaughton, 1983; Venter et al., 2021). In our study, we found higher R/S ratios of soybean plants under higher water availability. This indicated that the observed increase in water capture ability belowground in response to higher water availability contributes to the higher tolerance of soybean plants to parasitic plants.

### 4.2 Effect of P availability

Phosphorus is an essential macronutrient that is vital for plant growth and involved with leaf pigments and leaf photosynthetic enzymes (Verlinden et al., 2022). Although studies on the direct effect of P resources on the growth of host plants under parasitic plant stress are scarce, Yang et al. (2015) found that fertilization increased the biomass of parasitic *C. australis* and caused a more deleterious effect on the invasive host *Bidens pilosa*. In our study, soybean plants under higher P availability grew more rapidly (**Figures S1C**, **7**), supporting decreased (under W1 treatment) or unchanged (under W3 treatment) biomass of parasitic *C. australis* (**Figure 3B**) and also more damage to host plants caused by parasitic plants. These findings are consistent with both the growth rate model (GRM), which predicts lower tolerance of herbivore damage in higher-resource environments (Hilbert et al., 1981), and the plant stress hypothesis (White, 1969; Pardo and Pulido, 2017).

These results indicated that P and water availability similarly affected the growth of parasitic *C. australis*, but differently affected the tolerance of soybean plants to parasitism. Based on the limiting resource model (LRM), which posits that the level of tolerance is affected by the importance of the limiting resource and how herbivory affects the acquisition of the resource (Wise and Abrahamson, 2007; Hernan et al., 2019), we speculated that lower P but not lower water availability can induce compensatory growth of soybean plants in response to parasitism. Additionally, as there were significant interactive effects between water and P availability on *C. australis* biomass and the deleterious effect to soybean plants, the effect of P and water resources on the tolerance of soybean can be interdependent. In our study, we found that as P availability increased, the R/S ratio decreased, in contrast with the effect of increased water availability under no parasitism or low-intensity parasitism; the R/S ratio increased with water availability, and under high-intensity parasitism, the R/S ratio was less changed by increased water availability and was higher than that under no parasitism or low-intensity parasitism (**Figure 2A**). Previous studies showed that plants alter the R/S ratio to cope with resource limitations (Bonifas and Lindquist, 2006; Mašková and Herben, 2018). A large root system is more advantageous to the plant than a small root system for acquiring resources (Ma et al., 2010; Zhang et al., 2021). Therefore, increasing biomass allocation to roots under P stress may help soybean obtain more resources from the soil, which may alleviate the deleterious effect on soybean. Although the higher R/S ratio under drought conditions has also been widely reported (Kou et al., 2022), we found a low R/S ratio under low water availability. We speculated that this may be due to the continuous investment in root growth could penalize the shoot growth of soybean. Hence rather than growing a large root system in response to drought, soybean may allow the decreased resource allocation to roots for shoot growth (Whitmore and Whalley, 2009; Kou et al., 2022). Therefore, in our study, the different responses of the R/S ratio to water and P availability may result in the different deleterious effects of parasitism on soybean plants (**Figures S3B, C**).

### 4.3 Effect of parasitism intensity

When plants are damaged, plants do not just passively endure harm, as they have evolved adaptive mechanisms to compensate for such damage by increasing reproduction or regeneration (Lei et al., 2005; Li et al., 2008; Zhang et al., 2012; Peng et al., 2023). Zhang et al. (2012) found that infection with parasitic plants also caused compensatory growth in host plants, though this compensatory growth effect was modest. Such a compensatory effect was also found in response to *Cistanche deserticola* in its host *Haloxylon ammodendron* (Tan et al., 2004). In the present study, we found that the deleterious effect on soybean caused by parasitism was negatively related to *C. australis* biomass, but such correlation was significant only under high-intensity parasitism, which was not significant under low-intensity parasitism. This might be due to the different intensities of the compensatory growth induced by the strength of parasitism. Thus, the compensatory growth of host soybean plants induced by high-intensity parasitism of *C. australis* may have been insufficient to cover the loss of host plant biomass. In this study, the R/S ratio of soybean plants was significantly higher when soybean plants were under high-intensity parasitism (C2) than under no parasitism or low-intensity parasitism, indicating the ability of roots to capture resources plays an important role in the compensatory growth of host soybean plants caused by different parasitism levels.

### 4.4 Interactive effects among water availability, P availability, and parasitism intensity

Yet, there were a few studies have tested the individual effect of water or P supply, and the parasitism intensity on the tolerance of the host to parasitism. For example, Li et al. (2014) found that high-intensity parasitism led to a higher biomass decrease of the host *Mikania micrantha* than low-intensity parasitism, which is consistent with our results (**Figure 1B**). Yang et al. (2015) showed that fertilization caused a more deleterious effect on the invasive host *Bidens pilosa*, which is different from our results as we found that the effect of P supply on the tolerance of soybean to parasitism can be changed with water availability. Therefore, we suggest that more factors should be taken into account when evaluating the response of the host to parasitism.

A plant’s response to herbivory is plastic and varies according to the conditions it experiences. The effects of herbivory are governed by interactions between the environment and the affected plant (McNaughton, 1986), and plant responses vary according to prevalent biotic and abiotic conditions (Maschinski and Whitham, 1989; Bröcher et al., 2023). Plants adapt to drought and P deficiency by reducing growth (Høgh-Jensen et al., 2002; Wang et al., 2018). In this study, the SEM results showed that both P and water availability directly increased soybean plant biomass, and P availability and parasitism indirectly increased *C. australis* biomass (**Figure 7**). For example, under lower water availability, *C. australis* biomass decreased as P availability increased, while under higher water availability, *C. australis* biomass changed less as P availability increased. In addition, we also found that *C. australis* caused more damage to soybean hosts under higher P availability and lower water availability. These results indicated that the positive or negative effects of P availability on the response of soybean hosts to parasitism depended on the water availability. Additionally, the regression results showed that although drought increased the deleterious effect of parasitism on soybean plants, and this impact varied with P availability (**Figure 6**). The increase amplitude induced by drought was significantly higher when P availability was 5 μM compared to 10 μM (**Figure 6**). Many studies have shown that drought can amplify the effect of P deficiency by reducing the uptake of P, lowering the P concentration in cytoplasm, and reducing the ATP level in the chloroplast matrix (Tezara et al., 1999; Radersma et al., 2005; Diaz et al., 2018; Pereira et al., 2023). In contrast, P application can alleviate the stress effect of drought by enhancing photosynthesis through increasing the content of chlorophyll, intercellular CO_2_ concentration, and leaf area (Tariq et al., 2017; Wu et al., 2018). The possible mechanisms of the synergetic effect between P availability and water availability might be owing to the complementary responses of root resource capture ability increased by water availability and of leaf resource capture ability increased by P availability (Kerbiriou et al., 2013; Tariq et al., 2017; Wu et al., 2018). In this study, we also found that the optimal growth condition was 45–55% WHC and 15 μM P. Moreover, there was no significant difference in total biomass of soybean plants when the P supply was 10, 15, and 20 μM. This lack of an effect might be owing to P saturation. For soybean plants, the optimal P concentration was 10–15 μM, and no responses were observed when the concentration exceeded 20 μM.

Previous studies showed that parasitism intensity can affect the performance of the host plant (Li et al., 2014; Barath, 2021). Here, we found that there were interactive effects between parasitism level and water availability on the R/S ratio of soybean hosts. Under no parasitism or low-intensity parasitism, the R/S ratio increased with water availability, while under strong parasitism, the R/S ratio was lowest under intermediate water availability (**Figure 2**), indicating that the effects of both P and water availabilities on the response of soybean to parasitism depend on the parasitism intensity.

## 5 Conclusion

In this study, we found that water and P availability posed different effects on the tolerance of soybean to *C. australis* parasitism, but these effects were impacted by parasitism intensity. In general, lower water availability and high-intensity parasitism caused more damage to soybean plants and decreased the tolerance of soybean to parasitism. With the increasing P availability, the tolerance of soybean to parasitism fluctuated. *C. australis* caused the highest damage to soybean hosts under 5 μM P supply, 5–15% WHC, and high-intensity parasitism. We speculated the low tolerance of soybean under water stress may be due to the low R/S ratio, which may further decrease water acquisition of soybean from soil and thereby intensify the deleterious effect on soybean. The fluctuated tolerance with the increasing P availability may be due to the trade-off of resource allocation to root between water and P stress. These results provide basic empirical references for the control of parasitic weeds by managing irrigation and fertilizer application. We suggest that adequate water supply and moderate P supply may decrease the damage to soybean caused by *C. australis* parasitism in agriculture managements. Besides, although we only used one genotype of soybean as the host plant, we provided a solid example of how the host plant responded to parasitism under various conditions of water and P availability. Moreover, we encourage the inclusion of more genotypes of soybean and more host plant species to test our conclusions in future studies.

## Data availability statement

The raw data supporting the conclusions of this article will be made available by the authors, without undue reservation.

## Author contributions

YY: Writing - original draft and manuscript revision, and analysed the data. FG: conceived the ideas, designed the experiments, and performed the experiments. FY: improved the manuscript. JL: conceived the ideas and designed the experiments. M-HL: improved the manuscript. All authors contributed to the article and approved the submitted version.
