# Supplementary material for: Resource availability and parasitism intensity influence the response of soybean to the parasitic plant Cuscuta australis
Source: Front Plant Sci. 2023 May 9;14:1177154. doi: 10.3389/fpls.2023.1177154 (PMC10203557; doi:10.3389/fpls.2023.1177154)

**Fig. S1.** Mean (±SE) total biomass of soybean host plants. The panels show significant effects of (A) the main effect of parasitism by *Cuscuta australis*; (B) the main effect of water availability across three levels; (C) the main effect of P availability across five levels. C0, C1, and C2 indicate no parasitism and low- and high-intensity parasitism by *C. australis*, respectively. W1, W2, and W3 indicate that soil water content was 5–15%, 45–55%, and 85–95% water holding capacity, respectively. P0, P5, P10, P15, and P20 indicate P availability levels of 0, 5, 10, 15, and 20 μM, respectively.


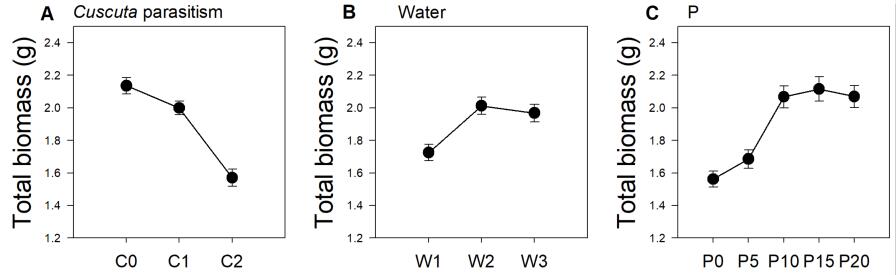


**Fig. S2.** Mean (±SE) the root/shoot (R/S) ratio of soybean host plants. The panels show significant effects of (A) the main effect of parasitism by *Cuscuta australis*; (B) the main effect of water availability across three levels. C0, C1, and C2 indicate no parasitism and low- and high-intensity parasitism by *C. australis*, respectively. W1, W2, and W3 indicate that soil water content was 5–15%, 45–55%, and 85–95% water holding capacity, respectively.


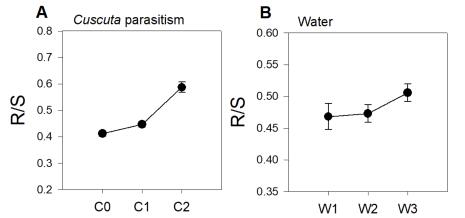


**Fig. S3.** Mean (±SE) deleterious effect of *Cuscuta australis* on soybean host plants. The panels show significant effects of (A) the main effect of parasitism by *Cuscuta australis*; (B) the main effect of water availability across three levels; (C) the main effect of P availability across five levels. C1 and C2 indicate low- and high-intensity parasitism by *C. australis*, respectively. W1, W2, and W3 indicate that soil water content was 5–15%, 45–55%, and 85–95% water holding capacity, respectively. P0, P5, P10, P15, and P20 indicate P availability levels of 0, 5, 10, 15, and 20 μM, respectively.


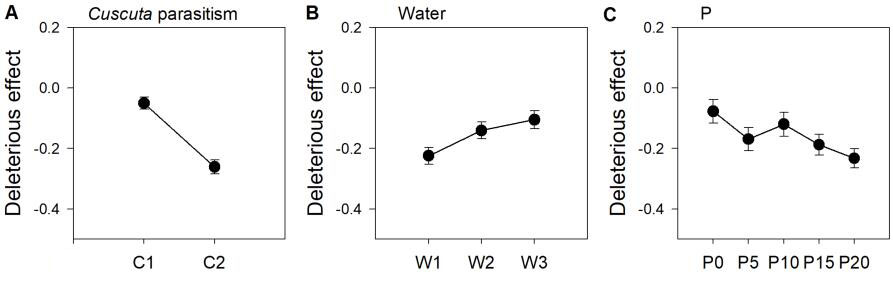

Supplement: Supplementary file 1 [file DataSheet_1.docx]
